# Draft Genome Sequence of Micromonospora sp. Strain WMMA1996, a Marine Sponge-Associated Bacterium

**DOI:** 10.1128/genomeA.00077-18

**Published:** 2018-02-22

**Authors:** Doug R. Braun, Marc G. Chevrette, Deepa D. Acharya, Cameron R. Currie, Scott R. Rajski, Tim S. Bugni

**Affiliations:** a Pharmaceutical Sciences Division, University of Wisconsin—Madison, Madison, Wisconsin, USA; b Department of Bacteriology, University of Wisconsin—Madison, Madison, Wisconsin, USA; c Laboratory of Genetics, University of Wisconsin—Madison, Madison, Wisconsin, USA

## Abstract

Micromonospora sp. strain WMMA1996 was isolated in 2013 off the coast of the Florida Keys, United States, from a marine sponge as part of bacterial coculture-based drug discovery initiatives. Analysis of the ∼6.44-Mb genome reveals this microbe’s potential role in the discovery of new drugs.

## GENOME ANNOUNCEMENT

Members of the genus Micromonospora are important producers of antibiotics, having yielded more than 700 compounds of medical value over the years (1). Compounds such as the aminoglycoside antibiotics gentamicin (2, 3) and netilmicin (2), the antitumor antibiotics lomaiviticins (4–7) and tetrocarcins (8, 9), the anthracycline antibiotics (10–12), and the enediyne calicheamicin (13–15) highlight both the structural diversity and medicinal impact of Micromonospora-derived natural products. In addition to compounds with clear antimicrobial or anticancer activities, members of the Micromonospora have produced natural products such as juvenimicin C (16), an activator of phase II detoxifying enzymes with cancer chemopreventive activities, and diazepinomicin, a farnesylated dibenzodiazepine with antioxidant and antiproteolytic activities proposed to protect against an assortment of age-related diseases such as diabetes, atherosclerosis, and various cancers (17).

In addition to serving as important “stand-alone” producers of natural products beneficial to human health, the Micromonospora have important applications within coculture systems that have begun to emerge. Coculturing of microorganisms has proven an effective means of activating otherwise dormant biosynthetic gene clusters (BGCs) to generate otherwise unattainable natural products. For instance, we recently discovered the novel antimicrobial agent keyicin using a Micromonospora sp. WMMB285/Rhodococcus sp. WMMA185 coculture system; the Micromonospora sp. proved to be the keyicin producer (18). In a similar fashion, metabolomics analyses of Dietzia sp. WMMA184/Micromonospora sp. WMMA1996 cocultures revealed Dietzia-dependent production of a number of small polyketide natural products by the Micromonospora sp. Despite such advances, and the clear historical importance of Micromonospora in drug discovery, little genomic information is available for these microbes relative to other actinomycetes. In contrast, the life cycle traits and habitats of these organisms and their diverse applications (most recently focused on biofuel production) have been rigorously investigated (19).

Micromonospora sp. strain WMMA1996 was isolated in 2013 from a marine-associated sponge (Tedania sp.) collected off the coast of the Florida Keys, United States, and its complete genome was sequenced by the University of Washington PacBio Sequencing Service using PacBio RS II (Pacific Biosciences) technology. Reads were constructed into a total of 10 contigs using the Canu v. 1.4 assembler; associated contigs ranged in size from 8 kb to 4.1 Mb (20). Open reading frames were predicted by Prodigal (21) and annotated using HMMer models for the TIGRfam (22), KEGG (23), and PFAM (21, 22) databases. The genome has 73.76% GC content. The organism's secondary metabolic content and potential were assessed on the basis of Anti-SMASH 4.0 (24) and PRISM (25). The Micromonospora sp. WMMA1996 genome was found to contain, but not be limited to, one type I polyketide (PKS), two type II PKS, one type III PKS, one nonribosomal peptide synthetase (NRPS) system, two type I PKS-NRPS hybrids, and four terpene biosynthetic gene clusters. The wealth of biosynthetic diversity housed within the Micromonospora WMMA1996 genome is unsurprising in light of our metabolomics analyses of Dietzia sp. WMMA184/Micromonospora sp. WMMA1996 cocultures, which revealed that the production of low-molecular weight (MW) polyketides is induced in coculture.

### Accession number(s).

The complete genome of Micromonospora sp. WMMA1996 has been deposited at the DDBJ/EMBL/GenBank under the project accession number PDHU00000000, which correlates to Bioproject number PRJNA407783.
